# Urease inhibitory activities of β-boswellic acid derivatives

**DOI:** 10.1186/2008-2231-21-2

**Published:** 2013-01-02

**Authors:** Sanaz Golbabaei, Roya Bazl, Sahand Golestanian, Farzaneh Nabati, Zinat Bahrampour Omrany, Behnam Yousefi, Reza Hajiaghaee, Shamsali Rezazadeh, Massoud Amanlou

**Affiliations:** 1 Department of Medicinal Chemistry, Faculty of Pharmacy and Medicinal Plants Research Center, Tehran University of Medical Sciences, Tehran, Iran; 2 School of Advanced Medical Technologies, Tehran University of Medical Sciences, Tehran, Iran; 3 Pharmacognosy & Pharmaceutics Department of Medicinal Plants Research Center, Institute of Medicinal Plants, ACECR, Karaj, Iran

**Keywords:** *Boswellia carterii*, Urease inhibitor, Boswellic acid, Docking, Autodock

## Abstract

**Background and the purpose of the study:**

*Boswellia carterii have* been used in traditional medicine for many years for management different gastrointestinal disorders. In this study, we wish to report urease inhibitory activity of four isolated compound of boswellic acid derivative.

**Methods:**

4 pentacyclic triterpenoid acids were isolated from *Boswellia carterii* and identified by NMR and Mass spectroscopic analysis (compounds **1**, 3-O-acetyl-9,11-dehydro-β-boswellic acid; **2**, 3-O-acetyl-11-hydroxy-β-boswellic acid; **3**. 3-O- acetyl-11-keto-β-boswellic acid and **4**, 11-keto-β-boswellic acid. Their inhibitory activity on Jack bean urease were evaluated. Docking and pharmacophore analysis using AutoDock 4.2 and Ligandscout 3.03 programs were also performed to explain possible mechanism of interaction between isolated compounds and urease enzyme.

**Results:**

It was found that compound **1** has the strongest inhibitory activity against Jack bean urease (IC_50_ = 6.27 ± 0.03 μM), compared with thiourea as a standard inhibitor (IC_50_ = 21.1 ± 0.3 μM).

**Conclusion:**

The inhibition potency is probably due to the formation of appropriate hydrogen bonds and hydrophobic interactions between the investigated compounds and urease enzyme active site and confirms its traditional usage.

## Introduction

Inhibition of enzymes by natural products which are proven to be the source of therapeutic agents, have increasingly attracted the interests of scientists as a valuable strategy in drug discovery. *Boswellia carterii* is widely distributed in the Indian subcontinent and Africa. The gum exudates of *Boswellia spp*. comprise of several pentacyclic triterpenoid acids, which are known as boswellic acids (BA’s) and reported to have different biological activities. Their ability to induce apoptosis choose them as potent compounds in treatment of colon, prostate and breast cancers [1-7] and a broad range of neurodegenerative conditions such as Alzheimer’s and Parkinson’s disease [8-11]. Also their inhibitory effect through topoisomerase I and II has been reported [12].

In addition, a number of extracted organic acids of this plant show other biological activities such as inhibition of peptic ulcer formation, probably by increasing the gastric mucosal resistance which its exact molecular mechanism is still not clear. In the recent report, it was shown that methanolic extract of *B*. *carterii* has urease inhibition activity [13,14].

Enzyme inhibition has already led to the discovery of the wide variety of useful drugs in the treatment of several diseases. It is found that *Helicobacter pylori* infection is the main cause of gastritis, peptic ulcer disease and gastric cancers [15,16].

Urease of *H*. *pylori* producing abundant amounts of ammonia (10–15% of total proteins by weight), make the growth and survival of bacteria possible, by increasing the pH of environment [17]. Therefore, it plays the major role in gastric diseases. Accordingly, urease inhibition has recently attracted much attention in pharmaceutical applications and discovery of potent anti-ulcer drugs. *H*. *pylori* has become resistant to many antibiotics, thus introducing new agents, like natural urease inhibitors, is of special importance. In addition, urease activity not only participates in the formation of kidney stones [18], but also involves in the development of urolithiasis, pyelonephritis, hepatic encephalopathy [19].

Throughout our work to search for natural urease inhibitor’s compounds from medicinal plants, we now report the isolation of four derivatives of boswellic acids to test their inhibitory activities through Jack bean urease. We employed Jack bean urease instead of bacteria’s one because it was previously found out that the mechanism and kinetics of inhibition for bacteria urease and Jack bean urease are comparable [20].

Molecular docking and simulation studies improve the reliability, accuracy of biological test, and show possible interactions between molecules and their target receptors. So the extracted compounds were subjected to molecular docking for better recognition of their interaction with urease.

## Material and methods

### Materials

Jack bean urease (EC 3.5.1.5 from Fluka Co, Switzerland), thiourea and all other chemicals used were of analytical grade (Merck Co, Germany). All solutions were prepared in MilliQ (Millipore, USA) water.

### Preparation of extract

An earlier report claimed that acetyl-keto-β-boswellic acid could not be separated from a mixture of acetyl-β-boswellic acid and acetyl keto-β-boswellic acid by any of the chemical methods, such as ketal formation [21] or semi-carbazide formation. In another method, methanol was found to be the most appropriate solvent for extraction, and used for simultaneous quantitative estimation of major BA’s from *Boswellia spp*.

In this research, solvent-solvent extraction, thin-layer (TLC) and column chromatography were performed to isolate the main active constituent of *B*. *carterii*. A total of 10 g of oleo-gum resin was grounded. Of this, extraction performed in 100 ml methanol three times. After filtration, the extract was concentrated and solvent was removed. KOH 2% was added to separate the acidic fraction and extraction was performed by ethyl acetate five times. In the next step, the aqueous phase was acidified with HCl 2%, and previous step was repeated. Then the organic phase washed with distilled water to obtain fractions containing BA’s compounds. Liquid chromatography was used to obtain related derivatives in organic fraction. The organic layers were dried, filtered and concentrated under vacuum on a rotary evaporator. Liquid chromatography of 5 g of sample which dissolve in chloroform is performed on 100 g of silica gel 60 (0.063 - 0.2 mm). Solvent partitioning of the crude extracts was used to divide the extract into eight fractions with different polarities. The fractions were combined based on similarity of the chemical composition determined by TLC analysis.

**Figure 1:**
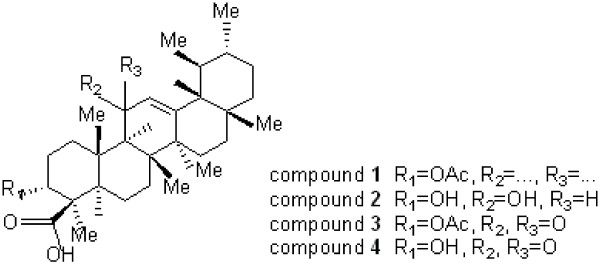
Structure of isolated boswellic acid derivatives.

Crude fractions 1-5 were concentrated under vacuum on the rotary evaporator. Fractions 6-7 were combined based on similarity of their TLC profile and subjected to further column chromatography and eluted with a methanol/chloroform gradient solvent mixture (3; 5; 7; 10 ml of methanol) to give fractions K, L, M and O. pure compound was identified using spectroscopic analysis which their structures are shown in Figure 1. The ^1^H and ^13^C-NMR spectra were recorded on a Bruker Avance 300-MHz NMR spectrophotometer, with internal standard tetramethylsilane (TMS). The structures were further confirmed by mass-spectral analysis and comparing with reported data for all the compounds.

### Biological evaluation

For urease inhibition assays after addition of phosphate buffer pH = 6.8, sonication was performed for 3 min, followed by centrifugation and evaluating absorbance of upper solution in λ = 278 nm which is attributed to the urease. By using following equation A = λ*bc*, where *c* is the concentration of solution (mol/L), *b* the Length of the UV cell, we can calculate the concentration of initially urease solution in this way. After appropriate dilution, the concentration of enzyme solution adjusted 2 mg/ml.

The assay mixture, containing 50 μl (2 mg/ml) of Jack-bean urease and 100 μl of different concentration of test compounds, which were dissolved in ethanol 20% previously, was added to 850 μl of 25mM urea and pre-incubated for 0.5 h in water bath at 37°C. The urease reaction was stopped after 30 min incubation by following procedure.

Urease activity was determined by measuring ammonia production using the indophenol method as described by Weatherburn [18]. After pre-incubation, 500 μl of phenol reagent (1% w/v phenol and 0.005% w/v sodium nitroprusside) and 500 μl of alkali reagent (1% w/v NaOH and 0.075% active chloride NaOCl) were added to 100 μl of incubation mixture and kept at 37°C for 30 min.

The absorbance was measured at 625 nm. All experiments were performed in triplicate in a final volume of 1 ml, and thiourea was used as a standard urease inhibitors. Percentage inhibitions were calculated using the formula (100 – (OD sample / OD control) × 100).

The concentration that provokes an inhibition halfway between the minimum and maximum response of each compound (relative IC_50_) was determined by monitoring the inhibition effect of various concentrations of compounds in the assay. The IC_50_ values were then calculated using GraphPad Prism 5 software.

### Molecular docking

The newly compounds were subjected for molecular docking. To validate observed activities and designing new inhibitors based on choosing the appropriate pharmacophore molecular docking was performed using AutoDock 4.2 along with AutoDockTools 1.4.5 using Lamarckian genetic algorithm on PDB structure (3LA4) http://www.pdb.org with resolution of 2°A [22].

In the present study, non-standard protein residues (KCX and CME) and the metal ions were included in the binding site specification. The docking calculations adopted in this work yield favorable orientations of ligands in a receptor. The graphical user interface ADT was employed to set up the enzymes: all hydrogen calculated and nonpolar hydrogens were merged to carbons. For determination of pharmacophore, Ligandscout 3.03 program was used [23].

Four potent inhibitors have sketched by Marvin sketch applet (Marvin package, Chemaxon company). Then adding polar hydrogens and rotatable bonds were done with Openbabel and AutodockTools, respectively [24,25]. Docking with a maximum number of 25 × 10^6^ energy evaluations were performed. After clustering analysis, conformation with the most favorable binding energy was selected.

## Result and discussion

Plants are the important source of new drug developments. Current study presents an investigation on urease inhibition of four isolated compounds from *B*. *carterii* which has been numbered as medicinal plant and had been used in stomach and colon disorders [26,27], on the other hand, detection of urease inhibitors, have been regarded as targets for new anti-ulcer drugs.

The aim of the present study is to confirm the possible anti-ulcer activity of the olibanum through inhibition of urease enzyme to support for its use as gastrointestinal agents in traditional medicine. Isolated compounds (Figure 1) were screened for urease inhibition activity against jack bean urease, and their relative IC_50_ values were depicted in Table 1.

**Table 1:** **Percent of inhibition, IC**_**50 **_**and ΔGº of four isolated boswellic acids derivatives**

**Compounds**	**Percent of inhibition (50 μg/ml)**	**IC**_**50**_ **± S.E.M (μM)**	**ΔGº (Kcal/mol)**
1	60.5	6.27 ± 0.037	−7.49
2	56.2	9.21 ± 0.069	−7.20
3	37.4	16.34 ± 0.063	−7.11
4	39.1	85.23 ± 0.065	−5.84
Thiourea	88.0	21.10 ± 0.3	−6.42

Out of four BA’s tested for their urease inhibitory activity, compound **4** was the only weakly active (IC_50_ = 85.23 ± 0.06 μM) and compound **1** (IC_50_ = 6.27 ± 0.03 μM) was found to be the most potent compound, compared to the standard inhibitor (thiourea, IC_50_ = 21.1 ± 0.3 μM). In this series, compounds **2** and **3** (IC_50_ = 9.21 ± 0.06, 16.34 ± 0.06 μM, respectively) are exhibited remarkable inhibition toward urease.

**Figure 2:**
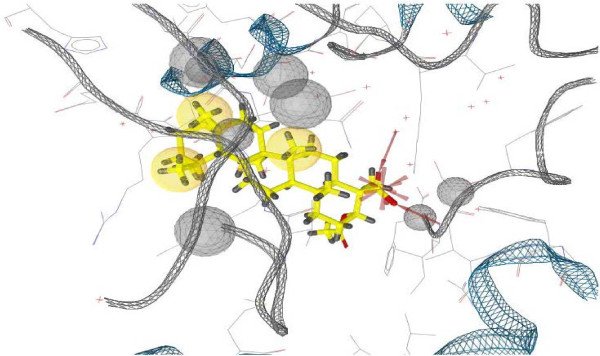
Molecular docking of compound 1, a 3D aspect.

It could be seen that the dehydro-substituted compound has stronger activity against urease than the hydroxy or keto-substituted ones. To investigate the binding effects between extracted BA’s and the Jack bean urease the molecular docking study was performed. The 3D binding model of compound **1** is depicted in Figure 2.

**Figure 3:**
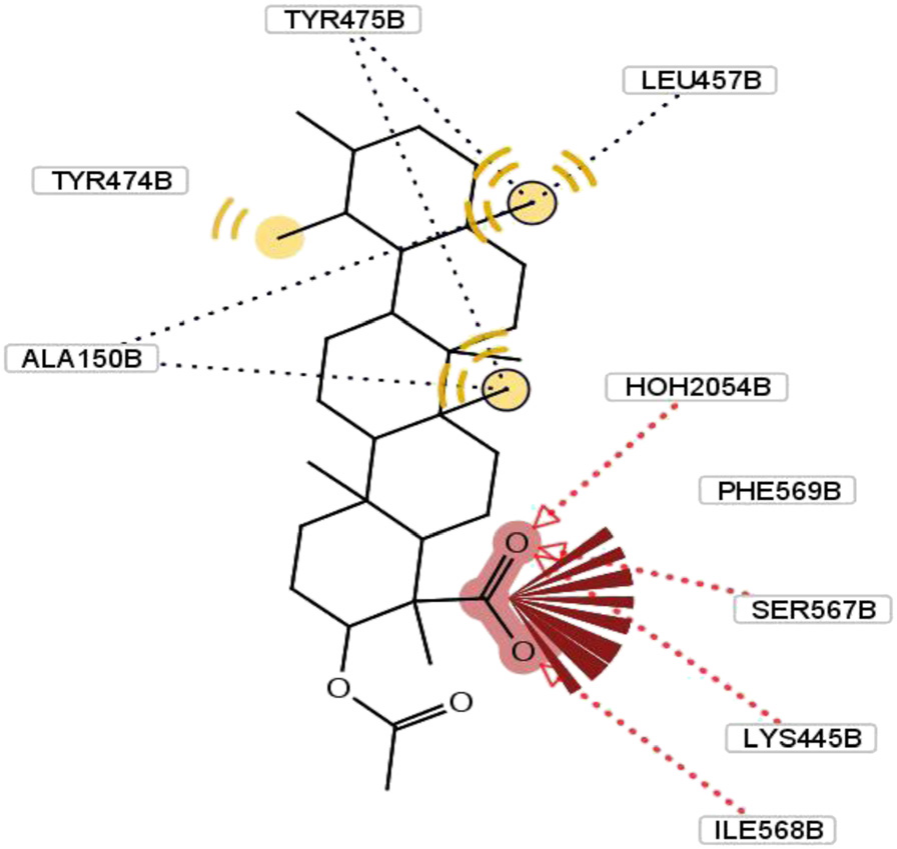
**Molecular docking of compound 1 and its interaction with different residue of urease enzyme active site involved in interaction. **Formation of hydrogen bonds between carbonyl of Phe 569, Ser 567, Ile 568 and Lys 445 from B-domain of enzyme and oxygen of the carboxyl moiety in compound **1**. Hydrophobic interactions with Ala 150, Leu 457, Try 474 and Try 475 of urease.

In the binding model, carbonyl of Phe 569, Ser 567, Ile 568 and Lys 445 from B-domain of enzyme forms hydrogen bonds with oxygen of the carboxyl moiety in compound **1**. Moreover, the compound may form hydrophobic interactions with Ala 150, Leu 457, Try 474 and Try 475 of urease (Figure 3). This potential inhibitory property possessed by this compound, may be attributed to the above hydrogen bonds and hydrophobic interactions.

**Figure 4:**
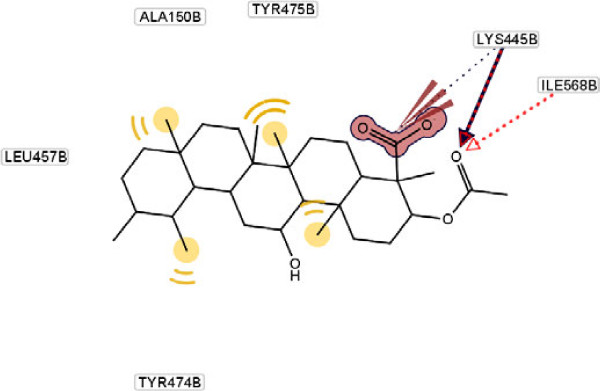
Molecular docking of compound 2 and its interaction with different residue of urease enzyme active site involved in interaction.

**Figure 5:**
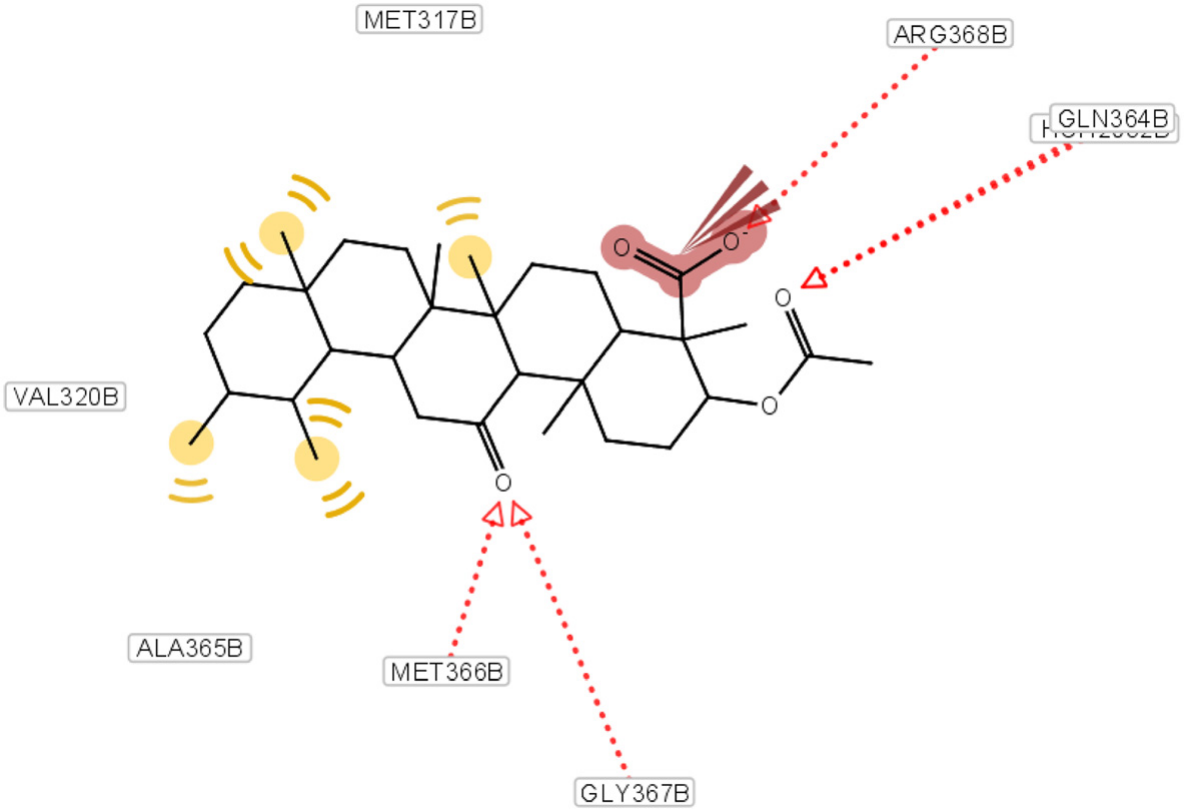
Molecular docking of compound 3 and its interaction with different residue of urease enzyme active site involved in interaction.

**Figure 6:**
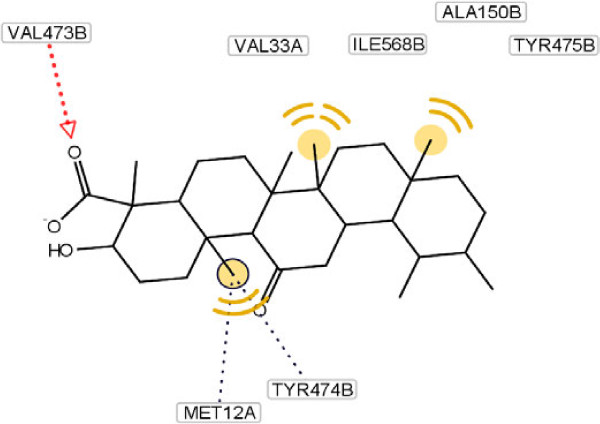
Molecular docking of compound 4 and its interaction with different residue of urease enzyme active site involved in interaction.

The docking calculations also reveal that the complex has the lower free energy of binding (-7.49 kcal/mol) compared with other extracted compounds, which may also explain the excellent inhibitory activity of it. As a comparison, a molecular docking study on the other compounds was also carried out, as it shown in Figures 4, 5, 6.

As it has shown, the enhanced percentage of urease inhibitory activity of compounds **1**, **2** and **4** with 60.5, 56.2 and 39.1%, respectively, compared to the structurally related compound **3** (37.4%) may either be due to the presence of methyl group and hydrophobic interaction of them with adjacent amino acids. This interaction becomes lower in the case of compound **3** because of steric factors. This phenomenon is depicted clearly in Figure 5.

As it has shown in Figure 5, for compound **3**, due to hydrogen bonding between oxygen of carboxyl group and Arg 368 and Gln 364, conformational locking happened, which possibly make the approach of the compound difficult towards the enzyme’s binding site and results in a sharp decline in inhibitory potential as compared to compound **4** (Figure 6). In another word, substitution at ortho position of C ring with different groups such as hydroxyl and ketone result to diminish of the inhibition potency. This effect explains the lower hydrophobic interaction between methyl group and amino acids present at interacting site.

Besides, presence of three kinds of hydrogen bonds formed by the carboxyl group of compound **1**, two kinds of hydrogen bonds in compound **2** and one kind in compound **4** with respective amino acids in the hydrophobic pocket can explain their percent of inhibition activity order against urease (Table 1). For compound **3**, despite the existence of four hydrogen bond’s percent of inhibition decrease dramatically. This observation can be explained by existing steric hindrance, which prevents the compound to interact with the hydrophobic site appropriately.

## Conclusion

In this research isolated compounds from *B*. *carterii* has been tested against urease, which may confirm its potential traditional use for stomach problems. Based on the above study, we can preliminarily conclude that the substituted C ring of BA’s may result to decreased inhibition concentration compared with the reference inhibitor thiourea. In addition, hydrophobic interactions between methyl group of compounds and near residues play an important role in the inhibition. This observation can be applied to the development of new antiulcer agents.

## Competing interest

There are no other conflicts of interest related to this publication.

## Authors’ contributions

All authors contributed to the concept and design, making and analysis of data, drafting, revising and final approval. MA is responsible for the study registration, financial and administrative support. SG, FN & ZBO are responsible for biological assays. RB, SaG & BY were involved for docking studies. RH & ShR were responsible for preparation of extract and isolation of compounds 1-4. SG, RB& MA were participated in data assembly and analysis, interpretation and manuscript writing. All authors read and approved the final manuscript.
